# Lysosomal damage after spinal cord injury causes accumulation of RIPK1 and RIPK3 proteins and potentiation of necroptosis

**DOI:** 10.1038/s41419-018-0469-1

**Published:** 2018-04-23

**Authors:** Shuo Liu, Yun Li, Harry M. C. Choi, Chinmoy Sarkar, Eugene Y. Koh, Junfang Wu, Marta M. Lipinski

**Affiliations:** 1 Department of Anesthesiology and the Center for Shock, Trauma and Anesthesiology Research (STAR), University of Maryland School of Medicine, Baltimore, MD USA; 2 Department of Orthopaedics, University of Maryland School of Medicine, Baltimore, MD USA

## Abstract

Necroptosis, a regulated necrosis pathway mediated by the receptor-interacting protein kinases 1 and 3 (RIPK1 and RIPK3), is induced following spinal cord injury (SCI) and thought to contribute to neuronal and glial cell death. However, mechanisms leading to activation of necroptosis after SCI remain unclear. We have previously shown that autophagy, a catabolic pathway facilitating degradation of cytoplasmic proteins and organelles in a lysosome-dependent manner, is inhibited following SCI in rats. Our current data confirm that inhibition of autophagy also occurs after thoracic contusive SCI in the mouse model, as indicated by accumulation of both the autophagosome marker, LC3-II and autophagy cargo protein, p62/SQSTM1. This was most pronounced in the ventral horn neurons and was caused by rapid inhibition of lysosomal function after SCI. Interestingly, RIPK1, RIPK3, and the necroptosis effector protein MLKL also rapidly accumulated after SCI and localized to neurons with disrupted autophagy, suggesting that these events may be related. To determine if lysosomal dysfunction could contribute to induction of necroptosis, we treated PC12 cells and primary rat cortical neurons with lysosomal inhibitors. This led to rapid accumulation of RIPK1 and RIPK3, confirming that they are normally degraded by the lysosomal pathway. In PC12 cells lysosomal inhibition also sensitized cells to necroptosis induced by tumor necrosis factor α (TNFα) and caspase inhibitor. Imaging studies confirmed that RIPK1 partially localized to lysosomes in both untreated and lysosomal inhibitor treated cells. Similarly, we detected presence of RIPK1, RIPK3 and MLKL in both cytosol and at lysosomes after SCI in vivo. Furthermore, stimulation of autophagy and lysosomal function with rapamycin treatment led to decreased accumulation of RIPK1 and attenuated cell death after SCI. These data suggest that lysosomal dysfunction after SCI may contribute to both inhibition of autophagy and sensitize cells to necroptosis by promoting RIPK1 and RIPK3 accumulation.

## Introduction

One of the features of secondary injury following traumatic spinal cord injury (SCI) is progressive neuronal cell death. The mechanisms of this death are diverse and include necrosis, classical apoptosis, as well as caspase-independent regulated cell death pathways^1^. Because many pathways are involved, attempts at improving SCI outcomes by inhibiting specific types of cell death have been largely unsuccessful^2^. This has led to more recent proposals for use of drugs that target upstream changes involved in the initiation of multiple cell death pathways. This, however, will require identification and characterization of the upstream cellular events involved in control of multiple pro-death pathways during SCI secondary injury.

Necroptosis is a form of regulated necrosis activated downstream of the tumor necrosis factor receptor 1 (TNFR1), dependent on the activity of the receptor-interacting protein kinase 1 (RIPK1) and 3 (RIPK3) and mediated by the mixed lineage pseudo-kinase MLKL^3,4^. The upstream mediator of necroptosis, RIPK1, is a key regulator of the innate immune responses involved in both inflammation and cell death, thus may represent an ideal target for reducing both cell death and inflammation in the central nervous system (CNS)^5^. Activation of necroptosis has been shown to contribute to cell loss and tissue damage in neurodegenerative diseases affecting the spinal cord, such as amyotrophic lateral sclerosis and multiple sclerosis^6,7^. Recent data also demonstrate involvement of necroptosis in neuronal and glial cell death after SCI^8–10^, and that its inhibition can improve functional recovery in animal models of SCI^10,11^. However, the mechanisms leading to activation of necroptosis after SCI and its relationship to other cellular pathways in this context remain unknown.

We recently demonstrated that contusive SCI in a rat model leads to inhibition of autophagy, a lysosome-dependent protein degradation pathway^12^. Inhibition of autophagy after SCI is particularly pronounced in the ventral horn motor neurons and contributes to endoplasmic reticulum (ER) stress induced apoptosis in these cells. Here we demonstrate that autophagy is similarly inhibited after SCI in a mouse model. Inhibition of autophagy flux is caused by a rapid decline in lysosomal function after injury, leading to accumulation of dysfunctional autophagosomes. Surprisingly, our data demonstrate that inhibition of lysosomal function also causes rapid accumulation of necroptosis mediators, RIPK1, RIPK3, and MLKL in neurons both in vitro and in vivo after SCI. These proteins accumulate both in the cytosol and at the lysosomes and can lead to cellular sensitization to necroptosis. Conversely, increasing function of the autophagy-lysosomal pathway in vivo can decrease necroptosis and general cell damage after SCI. These data point to a previously unexplored link between inhibition of the autophagy-lysosomal pathway and induction of neuronal necroptosis after SCI, and suggest improving lysosomal function as a potential multi-functional treatment after SCI.

## Results

### SCI leads to lysosomal dysfunction and inhibition of autophagy flux in mice

**Fig. 1:**
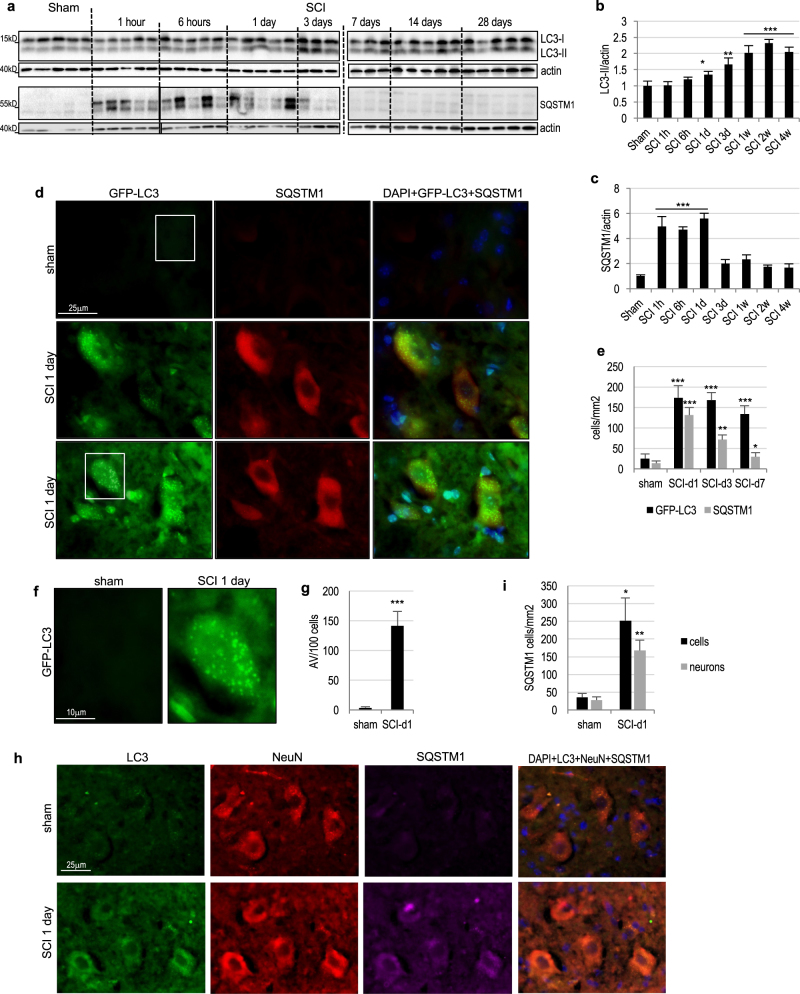
SCI leads to disruption of autophagy flux in ventral horn motor neurons. **a** Time course of accumulation of LC3-II and p62/SQSTM1 proteins in the spinal cord tissue surrounding injury site following thoracic contusion SCI in a mouse model. Each blot lane represents an individual animal. Full unedited western blots are presented in Supplemental Figure S1a. **b** Quantification of LC3-II levels from a and S1a. **c** Quantification of p62/SQSTM1 levels from a and S1a. *n* = 5. **d** IHC staining demonstrating accumulation of the GFP-LC3 autophagosome marker and p62/SQSTM1 in the same ventral horn cells 24 h after SCI in GFP-LC3 transgenic autophagy reporter mice. Images were acquired at 60 × magnification. Images for day 3 and 7 are presented in Supplemental Figure S2. **e** Quantification of GFP-LC3 and SQSTM1 data in d and S2a. *n* = 4. **f** Close-up of GFP-LC3 images from areas indicated in d showing accumulation of autophagosomes. **g** Quantification of autophagosomes/autophagic vesicles (AV) from data in d and f. *n* = 4 . **h** IHC images demonstrating that GFP-LC3 and p62/SQSTM1 accumulate primarily in neurons in the ventral horn at day 1 after SCI. Neurons were identified by staining with NeuN/RBFOX3. Images were acquired at ×20 magnification. **i** Quantification of data from h. *n* = 4 All data are presented as mean±SE. *p < 0.05, **p < 0.01, ***p < 0.001

We previously demonstrated that SCI leads to a temporary inhibition of autophagy in the rat contusion model^12^. To determine whether this is also the case in mice, we assessed accumulation of autophagosomes and autophagy flux after thoracic (T10) contusive injury^13^. Western blot data demonstrated progressive accumulation of the autophagy marker microtubule-associated protein 1A/1B light chain 3-II (LC3-II) in the spinal cord tissue surrounding the injury site starting by 6 h (Fig. 1a, b and Supplementary Figure S1a). This was accompanied by rapid accumulation of p62/SQSTM1 protein starting at 1 h after injury, indicating inhibition of autophagy flux^14^ (Fig. 1a, c). Interestingly, while increased LC3-II levels persisted up to 1 month after injury, levels of p62/SQSTM1 substantially declined within 1 week, indicating partial restoration of autophagy flux at later time points after SCI. These data were confirmed by immunohistochemistry (IHC) in *GFP-LC3* transgenic autophagy reporter mice^15,16^, demonstrating accumulation of GFP-LC3 and p62/SQSTM1 positive cells in injured spinal cord tissues (Fig. 1d, e). Consistent with the western blot data, GFP-LC3 positive cells persisted, while p62/SQSTM1 staining declined by 1 week after injury (Fig. 1e and Supplementary Figure S2). High-magnification imaging confirmed that GFP-LC3 localized to intracellular puncta corresponding to autophagosomes (Fig. 1f, g). Similar data were obtained by IHC staining with LC3 antibody in wild type mice (Fig. 1h). As in rat SCI^12^, the initial accumulation of autophagosomes and inhibition of autophagy flux at day 1 after SCI occurred primarily in neurons (Fig. 1h, i), with motor neurons of the ventral horn affected to the highest degree.

**Fig. 2:**
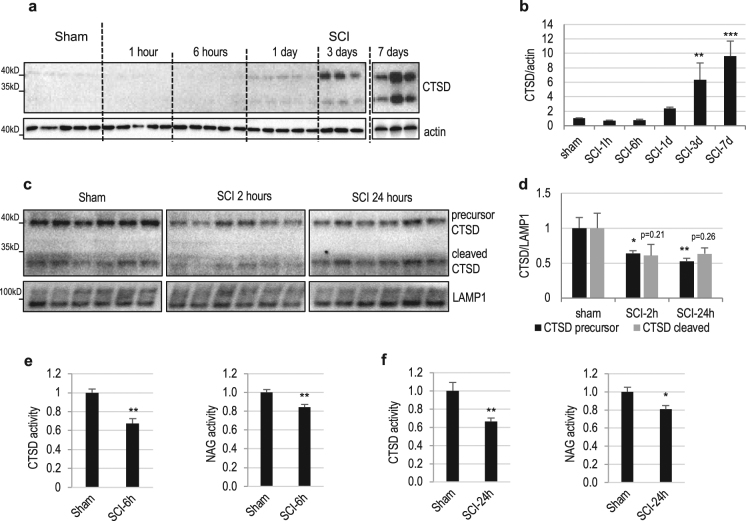
SCI leads to rapid lysosomal dysfunction in the spinal cord. **a** Time course of CTSD protein expression in spinal cord tissue surrounding injury site following SCI in mice. Full unedited western blots are presented in Supplemental Figure S1b. **b** Quantification of total CTSD data from **a** and S1b. *n* = 5. **c** Expression of CTSD in the lysosomes after SCI in mouse spinal cord. Sham and SCI mouse spinal cord tissue was fractionated to isolate lysosome-enriched fraction, then processed for western blot. Both full length precursor and cleaved active CTSD are indicated. Lysosomal membrane protein LAMP1 was used to identify lysosomal fraction and as a loading control. The exposure time is much longer than in panel a to allow better visualization of CTSD levels. Full unedited blots are presented in Supplemental Figure S3a. **d** Quantification of precursor and cleaved CTSD data from **c**. *n* = 6. **e**, **f** Activity of lysosomal enzymes CTSD and NAG was assessed in the spinal cord tissue surrounding injury site at 6 h (**e**) or 24 h (**f**) after SCI in mice. *n* = 6. All data are presented as mean±SE. *p < 0.05, **p < 0.01, ***p < 0.001

To determine the mechanisms leading to inhibition of autophagy flux we assessed lysosomal function after SCI. Consistent with previous reports^17^, levels of the lysosomal enzyme cathepsin D (CTSD) dramatically increased at late time points after injury (days 3 and 7). This reflects activation and proliferation of microglia and infiltrating macrophages after injury (data not shown)^12,16,18^. However, at early time points (1 and 6 h) after SCI we observed a slight decrease in levels of CTSD, indicating possible injury-induced decrease in lysosomal function (Fig. 2a, b and Supplementary Figure S1b). This is consistent with our previous rat data demonstrating defects in autophagosome-lysosome fusion after SCI^12^. To further investigate the initial decline in lysosomal function after SCI, we purified lysosome-enriched fractions^16^ from sham control and injured spinal cords at 2 and 24 h. We observed decrease in both precursor and cleaved CTSD protein levels at the lysosomes (Fig. 2c, d and Supplementary Figure S3a). Furthermore, enzymatic activity assay confirmed decreased CTSD activity at 6 and 24 h after SCI (Fig. 2e, f). Similar decline was observed for another lysosomal enzyme, N-acetyl-alpha-glucosaminidase (NAG) (Fig. 2e, f), consistent with our hypothesis that SCI leads to rapid decrease in lysosomal function, thus causing inhibition of autophagy flux.

### Autophagy/lysosomal damage correlates with markers of necroptosis in the injured spinal cord

**Fig. 3:**
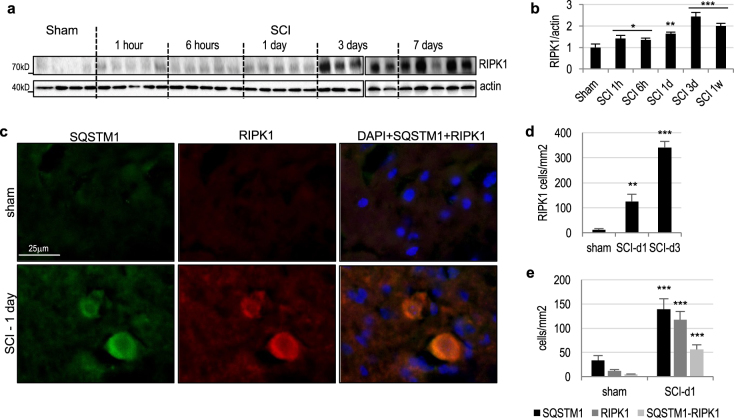
Expression of the necroptosis regulator RIPK1 correlates with inhibition of autophagy and lysosomal function after SCI. **a** The time course of RIPK1 protein expression in spinal cord tissue surrounding injury site following SCI in mice. Full unedited western blots are presented in Supplemental Figure S1c. **b** Quantification of RIPK1 data from **a** and S1c. *n* = 5. **c** IHC staining demonstrates accumulation of RIPK1 and p62/SQSTM1 in the same ventral horn cells at 24 h after SCI in mice. Images were acquired at ×20 magnification. Full time course images for RIPK1 are presented in Supplemental Figure S4a. **d** Quantification of RIPK1 data in **c** and S4a. *n* = 3–5. **e** Quantification of RIPK1 and SQSTM1 data in **c**. At day 1 after SCI 47.8% of RIPK1 positive cells were also positive for p62/SQSTM1. *n* = 8–13. All data are presented as mean±SE. *p < 0.05, **p < 0.01, ***p < 0.001

We previously demonstrated that inhibition of autophagy/lysosomal function after SCI leads to exacerbation of ER stress and contributes to neuronal apoptosis^12^. However, non-apoptotic cell death pathways are thought to also contribute to secondary injury after SCI^2^. To specifically assess contribution of necroptosis, we investigated expression of its upstream mediator RIPK1^3,4^. We observed rapid accumulation of RIPK1 in the spinal cord starting 1 h after SCI (Fig. 3a, b and Supplementary Figure S1c). This is consistent with the timing of lysosomal/autophagy inhibition and accumulation of p62/SQSTM1. Accumulation of RIPK1 protein was further confirmed by IHC (Fig. 3c, d and Supplementary Figure S4a), which also demonstrated that at 1day after SCI RIPK1 preferentially accumulated in neurons with inhibited autophagy/lysosomal function (p62/SQSTM1 positive, Fig. 3c, e). Therefore, accumulation of the essential necroptosis mediator RIPK1 occurs at the same time and in the same neuronal cells as SCI-induced defects in lysosomal function. Expression of RIPK1 protein increased further later after injury (days 3 and 7, Fig. 3a, b, d and Supplementary Figure S4a) but at these time points it was mainly localized to microglia and macrophages in the area surrounding injury site (data not shown), suggesting pro-inflammatory function^5^.

**Fig. 4:**
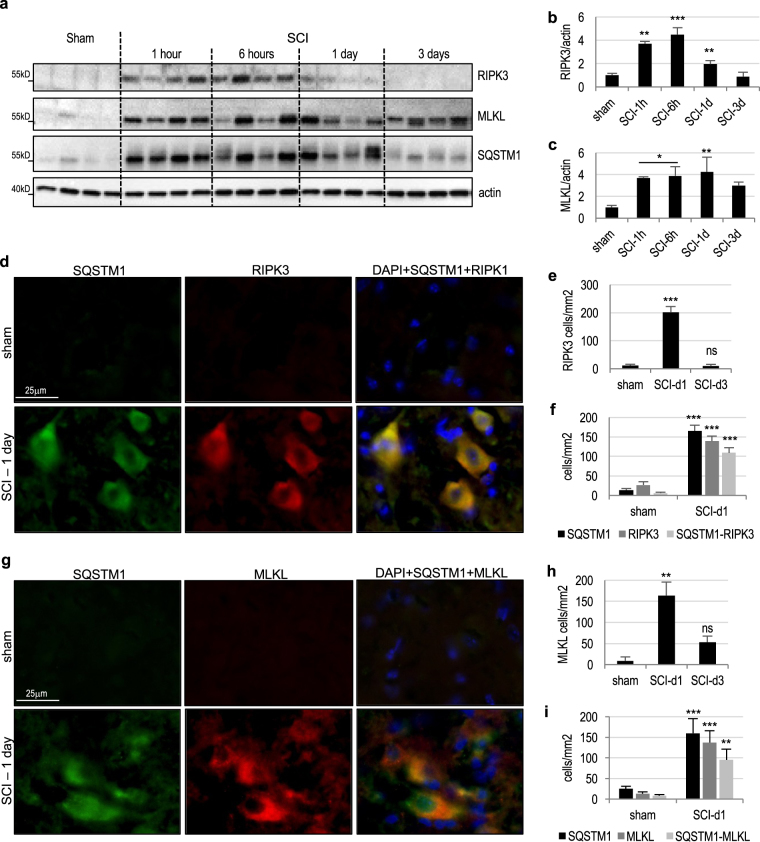
Expression of the downstream necroptosis mediators RIPK3 and MLKL correlates with inhibition of autophagy and lysosomal function after SCI. **a** The time course of RIPK3 and MLKL protein expression in spinal cord tissue surrounding injury site following SCI in mice. Full unedited western blots are presented in Supplemental Figure S5. **b** Quantification of RIPK3 data from **a**. **c** Quantification of MLKL data from **a**. *n* = 4. **d** IHC staining demonstrates accumulation of RIPK3 and p62/SQSTM1 in the same ventral horn cells at 24 h after SCI in mice. Images were acquired at ×20 magnification. Full time course images for RIPK3 are presented in Supplemental Figure S4b. **e** Quantification of RIPK3 data in **d** and S4b. *n* = 4. **f** Quantification of RIPK3 and SQSTM1 data in **d**. At day 1 after SCI 78.2% of RIPK3 positive cells were also positive for p62/SQSTM1. *n* = 12–17. **g** IHC staining demonstrates accumulation of MLKL and p62/SQSTM1 in the same ventral horn cells at 24 h after SCI in mice. Full time course images for MLKL are presented in Supplemental Figure S4c. **h** Quantification of MLKL data in g and S4c. *n* = 4. **i** Quantification of MLKL and SQSTM1 data in **g**. At day 1 after SCI 69.4% of MLKL positive cells were also positive for p62/SQSTM1. *n* = 10–12. All data are presented as mean±SE. *p < 0.05, **p < 0.01, ***p < 0.001

In addition to necroptosis RIPK1 is known to play a role in activation of NFκ-B and induction of inflammation, and in regulation of extrinsic apoptosis through caspase 8^5^. During necroptosis but not apoptosis or inflammation RIPK1 kinase is activated. This leads to recruitment and activation of necroptosis mediator RIPK3 and recruitment of the necroptosis executor, the mixed lineage pseudo-kinase, MLKL^19^. By 1 h after SCI we observed increase in RIPK3 and MLKL protein levels in the spinal cord (Fig. 4a–c and Supplementary Figure S5). Unlike RIPK1, levels of both proteins initially increased rapidly but declined by day 3 after injury, as expected for their function in cell death, which peaks around day 1 after SCI, rather than in later inflammation. The timing of RIPK3 and MLKL accumulation was further confirmed by IHC (Fig. 4d, e, g, h and Supplementary Figure S4b-c). To an extent even higher than RIPK1, RIPK3, and MLKL specifically accumulated in neurons with elevated p62/SQSTM1 protein (Fig. 4d, f, g, i). Therefore, lysosomal damage after SCI is associated with accumulation of RIPK1, RIPK3, and MLKL, suggesting potential link between lysosomal dysfunction and regulation of necrosome components in neurons.

### RIPK1/RIPK3 proteins are regulated by lysosomal degradation

**Fig. 5:**
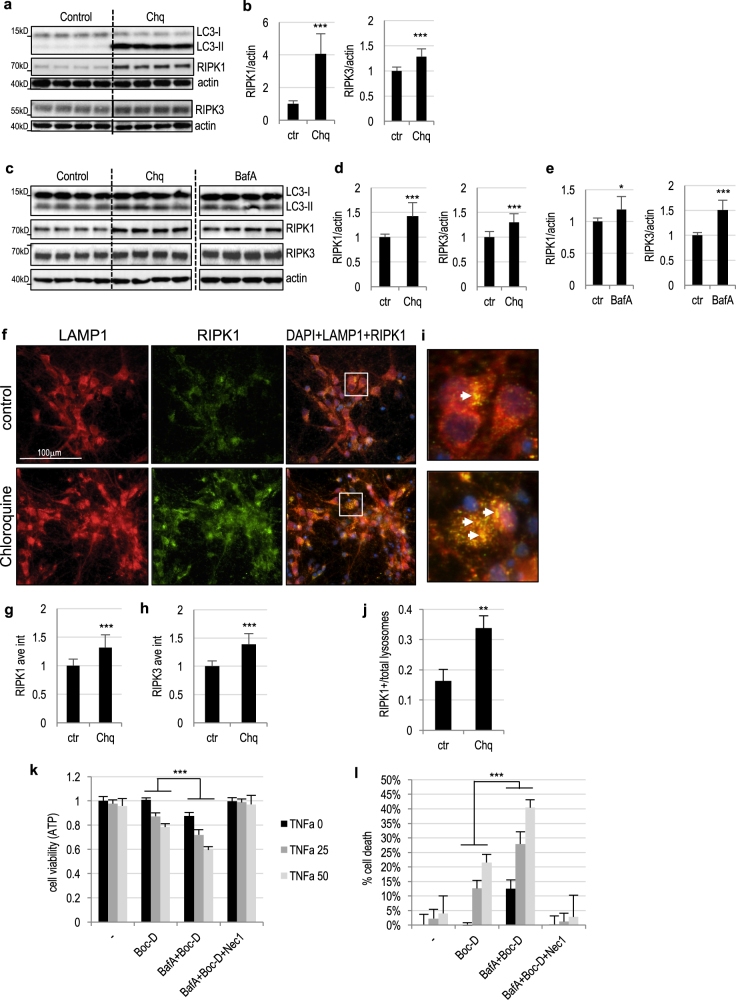
Lysosomal inhibition leads to accumulation of necroptosis mediators and sensitization to necroptosis in vitro. **a** Accumulation of RIPK1 and RIPK3 in PC12 cells following treatment with lysosomal inhibitor chloroquine (Chq, 100 μM). Cells were treated for 4 h. Full unedited western blots are presented in Supplemental Figure S6a-b. **b** Quantification of RIPK1 and RIPK3+/− Chq treatment data from a. *n* = 14–20. **c** Accumulation of RIPK1 and RIPK3 in rat cortical neurons following treatment with Chq or Bafilomycin A (BafA, 100 nM). Cells were treated for 4 h. Full unedited western blots are presented in Supplemental Figure S6d. **d** Quantification of RIPK1 and RIPK3+/− Chq treatment data from **c**. *n* = 12. **e** Quantification of RIPK1 and RIPK3+/− BafA treatment data from **c**. *n* = 8. **f** IF staining for RIPK1 in control and Chq treated rat cortical neurons. Cells were treated for 4 h; LAMP1 was used to visualize lysosomes. Images were acquired at ×20. **g** Quantification of RIPK1 intensity from **f**. *n* = 12. **h** Quantification of RIPK3 intensity in rat cortical neurons with and without Chq treatment. *n* = 8–9 Representative images are in Supplemental Figure S6e. **i** Close-up of the areas indicated in **f** showing colocalization of LAMP1 and RIPK1. Arrows point to RIPK1 positive lysosomes. **j** Quantification of RIPK1 positive lysosomes from **i**. *n* = 12. **k** Potentiation of necroptosis in PC12 cells treated with lysosomal inhibitor BafA. PC12 cells were treated with cycloheximide (20 μg/ml), pan-caspase inhibitor Boc-D (50 μM), and indicated doses of rat TNFα (0, 25, 50 ng/ml) to induce necroptosis in the presence or absence of BafA (100 nM). RIPK1 inhibitor necrostatin 1 (Nec1, 30 μM) was used to confirm that cell death was dependent on necroptosis. After 18 h cell viability was measured using luminescent ATP assay. *n* = 6 Additional controls are presented Supplemental Figure S7. **l** Data from **k** presented as % cell death. All data are presented as mean ± SD. *p < 0.05, **p < 0.01, ***p < 0.001

RIPK1 protein is regulated by ubiquitination, which affects both its function and stability^5^. RIPK1 protein stability has been thought to be regulated mainly by proteasomal degradation. However, a recent report indicated that under at least some circumstances both RIPK1 and RIPK3 may also be degraded via lysosome-dependent pathway^20^. To determine if lysosomal degradation may be important for RIPK1/RIPK3 expression in neural cells, we treated rat PC12 cells with lysosomal inhibitor, chloroquine (Chq)^14^. Four hour treatment led to inhibition of lysosomal function and autophagy flux and accumulation of both RIPK1 and RIPK3 proteins (Fig. 5a, b and Supplementary Figure S6a-b). Similar results were obtained when bafilomycin A (BafA) was used to inhibit lysosomal function (Supplementary Figure S6a, c). RIPK1 and RIPK3 also accumulated in primary rat cortical neurons treated with either Chq or BafA (Fig. 5c–e and Supplementary Figure S6d). Accumulation of RIPK1 and RIPK3 in Chq treated neurons was further confirmed by immunofluorescence (IF) staining (Fig. 5f–h and Supplementary Figure S6e). Image analysis also revealed partial co-localization of RIPK1 protein to LAMP1-positive lysosomes in both Chq treated and untreated cells (Fig. 5i, j). The degree of colocalization increased following Chq treatment indicating specific accumulation of RIPK1 in lysosomes (Fig. 5i, j). These data demonstrate that lysosomal localization and degradation may be involved in normal homeostasis of RIPK1 and RIPK3 proteins in neurons and that lysosomal dysfunction can lead to their accumulation.

### Lysosomal inhibition can sensitize cells to necroptosis

The fact that RIPK1 and RIPK3 are both regulated by lysosomal degradation, suggests that lysosomal function may also affect cellular sensitivity to necroptosis. To test this hypothesis, we treated PC12 cells with rat TNFα in the presence of pan-caspase inhibitor Boc-D to induce necroptosis. This led to dose-dependent cell death, which was potentiated in the presence of lysosomal inhibitor BafA and suppressed by necroptosis inhibitor, necrostatin 1^3^ (Nec1, Fig. 5k, l and Supplementary Figure S7). Interestingly, in the presence of BafA caspase inhibition alone was able to induce necroptotic cell death, even without TNFα treatment. This suggests that accumulation of RIPK1/3 upon lysosomal inhibition may be sufficient for necrosome formation and activation of necroptosis.

### Necroptosis mediators are present at the lysosomes in spinal cord in vivo

**Fig. 6:**
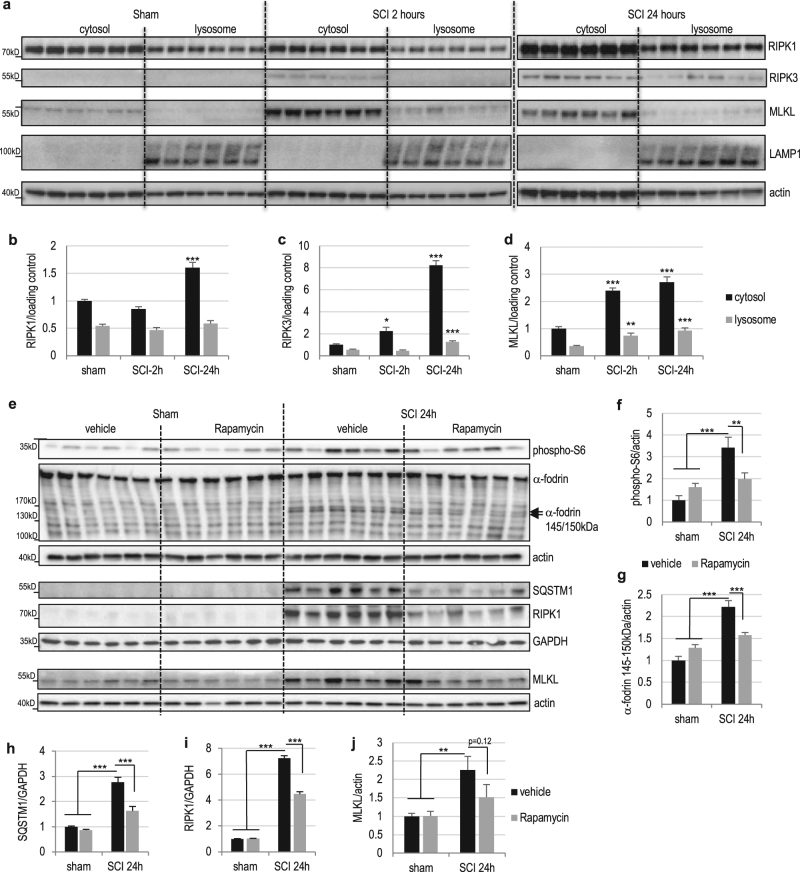
Lysosomal degradation contributes to regulation of necroptosis in vivo after SCI. **a** Expression of RIPK1, RIPK3, and MLKL in the cytosol and at the lysosomes in mouse spinal cord. Sham and SCI mouse spinal cord tissue was fractionated to isolate cytosolic and lysosome-enriched fractions. Lysosomal membrane protein LAMP1 was used to identify lysosomal fraction and as a loading control. Full unedited western blots are presented in Supplemental Figure S3b. **b** Quantification of RIPK1 data from **a** and S3b. **c** Quantification of RIPK3 data from **a** and S3b. **d** Quantification of MLKL data from **a** and S3b. *n* = 6. **e** Western blot data demonstrating that induction of autophagy and lysosomal function with Rapamycin decreased accumulation of RIPK1 and attenuated cell death in the mouse spinal cord tissue surrounding injury site at 24 h after SCI. Full unedited western blots are presented in Supplemental Figure S8a–c. **f** Quantification of phospho-S6 from **e** demonstrating inhibition of mTOR activity by Rapamycin after SCI. **g** Quantification of cleaved (145–150 kDa) α-fodrin from **e**. **h** Quantification of p62/SQSTM1 levels from **e**. **i** Quantification of RIPK1 levels from **e**. **j** Quantification of MLKL levels from **e**. *n* = 6. All data are presented as mean±SE. *p < 0.05, **p < 0.01, ***p < 0.001

To confirm that lysosomal dysfunction may be also contributing to regulation of necroptosis in vivo after SCI, we investigated intracellular localization of its mediators. Subcellular fractionation of spinal cord samples revealed that RIPK1, RIPK3, and MLKL were present and accumulated in both cytosolic and lysosome-enriched fractions of the spinal cord after SCI (Fig. 6a–d and Figure S3b), consistent with the hypothesis that they are regulated by lysosomal degradation and accumulate at lysosomes when degradation is blocked.

### Restoration of autophagy-lysosomal function attenuates necroptosis after SCI

To determine if improving autophagy-lysosomal function may decrease induction of necroptosis after SCI, we treated sham and injured mice with mTOR inhibitor, rapamycin, which has been previously shown to improve functional outcomes after SCI^21,22^. Inhibition of the mTOR pathway is known to both increase autophagy flux and induce lysosomal biogenesis, and we have previously demonstrated that it leads to increase in the number of lysosomes in vivo in the brain^16^. Similar to previous reports in experimental traumatic brain injury (TBI)^23^, we observed increased phosphorylation of mTOR target, the ribosomal protein S6, in the spinal cord tissue after SCI. Phospho-S6 was decreased in rapamycin-treated mice, confirming inhibition of mTOR (Fig. 6e, f and Supplementary Figure S8a). This was accompanied by decreased cleavage of α-fodrin in rapamycin-treated as compared to vehicle treated SCI mice, confirming previously reported decreased cell damage and death (Fig. 6e, g and Supplementary Figure S8b)^21,24^. Importantly, rapamycin treatment attenuated accumulation of p62/SQSTM1 at 24 h after SCI, indicating improvement in lysosomal function and autophagy flux (Fig. 6e, h and Supplementary Figure S8c). Consistent with the hypothesis that lysosomal dysfunction contributes to necroptosis, we also observed significant decrease in RIPK1 accumulation in rapamycin-treated SCI animals as compared to vehicle controls (Fig. 6e, i and Supplementary Figure S8c). Rapamycin also attenuated accumulation of MLKL after SCI, although this effect failed to reach significance due to one outlier in the treated group (Fig. 6e, j and Supplementary Figure S8d).

In addition to being directly involved in necrosome formation, RIPK1 has been implicated in regulation of autocrine production of TNFα through the AKT/mTOR/JNK pathway^25,26^. To determine if this pathway may also be affected, we assessed levels of AKT-Thr308 phosphorylation. Although we detected increase in phospho-AKT Thr308 after SCI, this was not altered by rapamycin treatment (Supplementary Figure S8a, e). Phosphorylation of JNK was also not affected by either SCI or rapamycin (Supplementary Figure S8b, f), suggesting that JNK pathway may not be essential for TNFα production at day 1 after SCI.

## Discussion

We previously demonstrated that autophagy flux is temporarily inhibited following thoracic SCI in a rat model and is associated with enhancement of neuronal ER stress and caspase 12/caspase 3 dependent apoptosis^12^. Our current data confirm that autophagy is similarly inhibited after SCI in mice. The initial rapid increase in both p62/SQSTM1 and LC3-II and subsequent normalization of p62/SQSTM1 but not LC3-II levels also suggest that similarly to rat, autophagy flux is initially inhibited after mouse SCI but eventually recovers. At this point we are not certain whether eventual recovery of autophagy flux is due to death of the initially affected neuronal cells or if some of them are able to recover. However, our data indicate that the initial inhibition of autophagy flux is caused by a rapid decline in lysosomal function observed after SCI, as indicated by decrease in both the protein levels and activity of lysosomal enzymes. The timing of the lysosomal dysfunction is very rapid—it is apparent as early as 1 h after injury. This suggests that lysosomal damage may be one of the apical cellular events contributing to initiation, early propagation and potentiation of the secondary injury cascades.

Consistent with the notion that lysosomal damage may specifically contribute to multiple mechanisms of neuronal cell death after SCI, in addition to the previously reported ER stress and apoptosis^12^, we observed accumulation of markers of necroptosis specifically in the neurons displaying signs of autophagy flux inhibition and lysosomal damage. Interestingly, these markers, RIPK1, RIPK3, and MLKL are expressed after SCI with different kinetics. Although accumulation of each protein starts within hours after injury, RIPK1 continues to increase over the period of a week, before gradually subsiding. This time course likely reflects RIPK1 involvement in both cell death and inflammatory processes^5^. The latter are at least in part mediated through complex I/RIPK1 dependent activation of NFκB downstream of TNFR1. Consistent with this notion, while the earliest (day 1) expression of RIPK1 occurred primarily in neurons, at later time points it localized to cells with activated microglia/macrophage morphology (data not shown). On the other hand, while RIPK3 and MLKL are required together with RIPK1 for necrosome formation and induction of necroptosis^19^, they are not involved in complex I/RIPK1 dependent NFκB signaling. Consistent with a function in neuronal necroptosis after SCI, both RIPK3 and MLKL accumulate rapidly in neurons and peak by day 1, the time of maximum cell death. Their levels decline by day 3. This is also consistent with the timing of lysosomal and autophagy dysfunction after SCI, which declines by day 3 and largely resolves within a week after injury.

It has been previously demonstrated that activation of necroptosis contributes to neuronal and glial cell death after SCI^8–10^ and that treatment with RIPK1 kinase inhibitor, necrostatin 1 (nec1), can improve cell survival and functional outcomes after injury^10,11^. However, the mechanisms leading to activation of necroptosis after SCI and its relationship to other cellular pathways are not clear. In particular, while accumulation of RIPK1, RIPK3, and MLKL after SCI has been noted and used as a marker of necroptosis^8^, the reasons and timing for the buildup of these proteins remained unknown. Necroptosis is activated following ligation of the TNFR1 receptor by TNFα and requires activation of RIPK1 and RIPK3 kinase activity and recruitment of MLKL to form necrosome^5,19^. No protein synthesis is necessary, and in fact, in many cell types necroptosis is specifically induced under conditions where protein translation is inhibited in order to suppress pro-survival function of NFκB^3^. Instead, RIPK1 protein recruitment to complex I versus complex II and necrosome is regulated by ubiquitination^5^. Ubiquitination also affects RIPK1 protein stability, which until recently has been thought to depend exclusively on proteasomal degradation. However, a recent report indicated that under at least some circumstances ubiquitinated RIPK1 and RIPK3 may be also degraded via lysosome-dependent pathway^20^. Consistent with the importance of this pathway in the CNS, our data suggest that lysosomal inhibition in vitro and in vivo specifically causes rapid accumulation of necroptosis mediators in neurons. Confirming the function of lysosomes in their degradation, RIPK1, RIPK3, and MLKL accumulate both in the cytosol and at the lysosomes. Therefore, our data support a model where under basal conditions lysosomal degradation keeps the levels of necroptosis mediators in neuronal cells low. When lysosomal function is impaired, such as after SCI, RIPK1, RIPK3, and MLKL accumulate, making the cells more sensitive to necroptosis.

Our in vitro experiments indicate that lysosomal inhibition can lead to induction of necroptosis even in the absence of TNFα. Therefore, it is possible, that following lysosomal inhibition accumulation of RIPK1, RIPK3 and MLKL per se can lead to the formation of necrosome and initiation of necroptosis. It has been previously suggested that under some circumstances formation of necrosomes can occur on the surface of dysfunctional autophagosomes through interaction with the autophagy protein, ATG5^27^. However, we were not able to detect interaction of necrosome components with ATG5 either in vitro or in vivo (data not shown). Furthermore, our in vitro IF experiments indicated that RIPK1 colocalized primarily with LAMP1-positive lysosomes rather than with autophagosomes (data not shown). However, we cannot exclude the possibility that in addition to directly leading to accumulation of necroptosis proteins, lysosomal dysfunction after SCI can further potentiate necroptosis after SCI by causing accumulation of autophagosomes and thus providing additional platform for necrosome assembly. These mechanisms are not mutually exclusive and further experiments will be necessary to clarify their respective contribution.

Our data point to the crucial role of lysosomal dysfunction as an early event contributing to propagation of secondary injury through induction of apoptosis and necroptosis. Consistently with the causative role of lysosomal dysfunction, treatment of SCI mice with the mTOR inhibitor and inducer of autophagy and lysosomal biogenesis, rapamycin, attenuated both inhibition of autophagy flux and accumulation of RIPK1. Treatment with rapamycin has been previously shown to confer neuroprotection and improve functional outcomes in rodent models of SCI^21,22,28^. However, the mechanisms of this protection remained unclear. Interestingly, combined inhibition of mTOR and AKT but not either treatment alone, has been shown to suppress necroptosis in cultured cortical neurons^29^ and provide neuroprotection in a mouse model of traumatic brain injury (TBI)^23^. Our data indicate that inhibition of mTOR is sufficient to at least partially attenuate necroptosis after SCI. In addition to being directly involved in necrosome formation, RIPK1 has been implicated in regulation of autocrine production of TNFα through the Akt/mTOR/JNK pathway^25,26^. We did not observe any changes in either AKT or JNK activation following treatment with only rapamycin. Therefore, similar to TBI, inhibition of both AKT and mTOR may be necessary to cause downregulation of JNK and autocrine production of TNFα. We hypothesize that combination of Akt inhibitor and rapamycin treatment after neurotrauma may affect necroptosis by both suppressing accumulation of RIPK1 and other necrosome components, and by decreasing autocrine TNFα production and activation of necrosome assembly downstream of TNFR1 ligation.

Together our data suggest that, lysosomal function may be an important upstream cellular event involved in control of multiple pro-death pathways during SCI secondary injury, including inhibition of autophagy flux, activation of ER stress dependent apoptosis and sensitization to necroptosis. Thus, restoring or enhancing lysosomal function may represent an interesting intervention avenue^28^. Inhibition of autophagy/lysosomal pathway and activation of necroptosis have been shown to contribute to cell loss and tissue damage in other CNS trauma models, including traumatic brain injury^16,23,30,31^. Necroptosis is also involved in neurodegenerative diseases affecting the spinal cord, such as amyotrophic lateral sclerosis and multiple sclerosis^6,7^. As most neurodegenerative disorders, these diseases are also associated with defects in the lysosomal/autophagy pathway. Therefore, lysosomal dysfunction may contribute to induction and/or sensitization to necroptosis also in these diseases.

## Materials and methods

### Contusive SCI in mice

Adult male C57BL/6J mice (8–10 weeks old, 20–25 g; Jackson Laboratory) or *GFP-LC3* autophagy reporter transgenic male mice^15^ were anesthetized with isoflurane and received T10 spinal contusions using the Infinite Horizon Spinal Cord Impactor (Precision Systems and Instrumentation) with a force of 60 kdyn, a moderate injury^13,32^. Bladders were manually expressed twice daily until a reflex bladder was established (7–14 d after SCI). The number of mice at various time points in each study is indicated in the figure legends. All procedures were performed under protocols approved by the University of Maryland School of Medicine Institutional Animal Care and Use Committee (IACUC).

### Drug treatments

Sham and SCI mice were assigned to a treatment group according to a randomized block experimental design. Rapamycin (Sigma-Aldrich, US, Cat. No. 37094) was dissolved in DMSO and then diluted in vehicle containing 0.25% PEG400 and 0.25% Tween 80. The final concentration of DMSO was adjusted to 0.1%. Rapamycin was injected twice (5 min and 4 h post injury) intraperitoneally in treatment group (*n* = 6) at a dose of 5 mg/kg based on prior investigation^16^. Mice of the control group (*n* = 6) were injected with the equivalent volume of vehicle. Animals were anesthetized and processed for western blot at 24 h after injury.

### Western blot analysis

Mouse spinal cord tissue (5 mm) centered on the injury site was lysed and homogenized in RIPA buffer (Sigma-Aldrich) supplemented with 1 × protease inhibitor cocktail (Sigma-Aldrich) and phosphatase inhibitor cocktail II and III (Sigma-Aldrich), sonicated and centrifuged at 20,000 × *g* for 20 min^12,32^. Protein concentration was determined by the Pierce BCA method (ThermoFisher Scientific, US). Samples were run on 4–20% SDS–PAGE (Bio-Rad, Hercules, CA), and transferred to PVDF membrane (Bio-Rad). Membranes were incubated with primary antibodies overnight, with HRP-conjugated secondary antibodies for 1 h, visualized using SuperSignal West Dura Extended Duration Substrate (ThermoFisher), and imaged with ChemIDoc TM MP system (Bio-Rad). The signal (optical density) was quantified by Image Lab software (Bio-Rad). Primary antibodies: LC3 (1:1000, Novus, Cat. No. NB100-2220), p62/SQSTM1 (1:1000; BD Bioscience, Cat. No. 610832), CTSD (1:100, Santa Cruz Biotechnology, Cat. No. sc-6486), RIPK1 (1:1000; Cell Signaling, Cat. No. 3493, Danvers, MA), RIPK3 (1:1000, ProSci, Cat. No. 2283, Poway, CA), MLKL (1:1000, EMD Millipore, Cat. No. MABC604, Temecula, CA), β-Actin (1:10 000; Sigma-Aldrich, Cat. No. A1978), Fordin/SPTAN1 (1:5000; Enzo Life Science International, Cat. No. BML-FG6090), GAPDH (1:2000; Millipore, Cat. No. AB2302), LAMP1 (1:1000; Cat. No. 1D4B, developed by J. Thomas August, Developmental Studies Hybridoma Bank/NICHD, maintained by The University of Iowa, Department of Biology, Iowa City, IA). Expression levels of the target protein bands were normalized to β-actin, GAPDH or LAMP1 (lysosomal fraction), and expressed as a fold of sham control.

### Subcellular fractionation

Around 5-mm fragments of spinal cord tissue centered on the injury site or corresponding site in sham animals were collected from sham mice, and at 2 or 24 h after injury and homogenized in ice-cold buffered solution containing 0.32 M Sucrose, 10 mM Hepes and protease and phosphatase inhibitors. Homogenates were centrifuged at 800 × *g* for 10 min at 4 °C to pellet down the nuclei. Supernatants were sequentially centrifuged at 20,000 × *g* for 20 min at 4 °C to pellet the heavy membrane/crude lysosomal fractions and at 100,000 × *g* for 1 h at 4 °C to pellet light membrane fractions^16^. Both supernatant and suspended pellet fractions were re-centrifuged to minimize cross contamination from the different subcellular fractions. All pellets were re-suspended in homogenization buffer. Protein concentration was estimated using BCA reagent; samples were analyzed by western blot.

### Lysosomal activity assays

Mice were anesthetized, perfused with ice-cold saline, and 5-mm spinal cord tissue surrounding the site of injury was dissected, homogenized in ice cold cell lysis buffer and centrifuged at 15,000 × *g* for 5 min at 4 °C. Protein concentration was estimated by the BCA method; 50 ng of protein was used per assay. The CTSD and NAG assays were performed using a fluorometric assay kits (Abcam, Cat. No. ab65302, Sigma-Aldrich, Cat. No. CS0780) as per the manufacturer’s instructions^16^. Fluorescence released from the synthetic substrate was measured using fluorescent plate reader (Synergy Hybrid, Biotek) at Ex/Em = 328/460 nm.

### Immunohistochemistry (IHC)

Animals were intracardially perfused with PBS, then with 4% paraformaldehyde^32,33^. A 1.0-cm segment of spinal cord centered at the injury epicenter was sectioned at 20 µm thickness and thaw-mounted onto Superfrost Plus slides (ThermoFisher). Sections were blocked in 5% goat or donkey serum in PBS/0.025% Triton X-100, incubated with primary antibodies overnight and with secondary antibodies for 1 h. Cell nuclei were labeled with 4′,6-diamidino-2-phenylindole (DAPI, Sigma-Aldrich), slides were cover-slipped with an anti-fading medium (Hydromount; National Diagnostics). Primary antibodies: LC3 (1:200, Novus, Cat. No. NB100-2220), p62/SQSTM1 (1:200; Progen, Cat. No. GP62-C, Heidelberg, Germany), CTSD (1:100, Santa Cruz Biotechnology, Cat. No. sc-6486), RIPK1 (1:100; Cell Signaling, Cat. No. 3493, Danvers, MA), RIPK3 (1:100, Enzo, Cat.No. ADI 905-242-100, Farmingdale, NY), MLKL (1:100, EMD Millipore, Cat. No. MABC604, Temecula, CA), NeuN/RBFOX3 (1:500; Millipore, Cat. No. MAB377). Secondary antibodies: alexa fluor 488 goat anti-rabbit (Cat. No. A11034), alexa fluor 546 goat anti-mouse (Cat. No. A11030), alexa fluor 568 goat anti-guinea pig (Cat. No. A11075), alexa fluor 633 goat anti-mouse (Cat. No. A21052) and alexa fluor 546 donkey anti-goat (Cat. No. A11056, all Invitrogen).

### Image acquisition and quantification

All images were acquired 0.5–1 mm rostral to the epicenter^33^. Images from ventral horns of gray matter were acquired using a fluorescent Nikon Ti-E inverted or Nikon Ni-E upright microscope, at ×20 (CFI Plan APO VC 20 × NA 0.75 WD 1 mm) or ×60 (CFI Plan APO VC 60 × NA 1.4 Oil) magnification^12,16^. All images for each data set were acquired using the same parameters (magnification, exposure time, gain, etc). All ×60 images were acquired as z-stacks and focused using Extended Depth of Focus (EDF) module of Elements software (Nikon). The background of each image was subtracted using background region of interest (ROI). All images were quantified in unbiased automated manner using custom macros in Elements: nuclei were identified using Spot Detection algorithm; cells positive for any of the immunofluorescence markers were identified using Detect Regional Maxima algorithm, followed by global thresholding. Number of positive cells was normalized to the total imaged ventral horn area (mm^2^). Intracellular puncta were detected using Spot Detection and normalized to the number of cells imaged. For each experiment data from all images from same region in each mouse was summed up and used for final statistical analysis. At least 500–1000 cells were quantified per mouse per experiment. All quantification was performed on original unedited images. For visualization purposes in figures only brightness and contrast were adjusted; all adjustments were applied to entire image area and equally to all panels in the same figure. In multi-color overlay images brightness of the DAPI channel was selectively decreased to allow better visualization of other channels.

### Tissue culture and treatments

Rat pheochromocytoma PC12 cells (ATCC, Cat. No. CRL-1721.1) were maintained in F-12K nutrient mixture (1 × ) Kaighn’s modification with 5% fetal bovine serum (FBS, Gibco, Cat. No.10082-147), 10% horse serum (Gibco), penicillin, and streptomycin^34^. Rat embryonic neurons were extracted from embryo of female spraque-dawley rats (E17, from Taconic) as described^35^. Neurons were maintained in neurobasal medium (Gibco, Cat. No. 12348-017) with B-27, L-glutamine, penicillin and streptomycin and allowed to differentiate for 1 week prior to experiments. For western blot analysis, PC12 cells were seeded in 24-well plates at a density of 5 × 10^4^ cells/well. After reaching 80% confluence (PC12 cells) or 1 week differentiation (neurons), cells were incubated with or without 100 μM chloroquine (Sigma-Aldrich, Cat. No. C6628) or 100 nM bafilomycin A (Sigma-Aldrich, Cat. No. B1793) for 4 h^14^, lysed in homogenizing buffer and analyzed by western blot. For immunofluorescence (IF) imaging cells were seeded on cover slips, treated as above, then fixed in 2% paraformaldehyde. Coverslips were blocked in 5% goat or donkey serum in PBS/0.025% Triton X-100, incubated with primary antibodies (same as for IHC) overnight and with secondary antibodies for 1 h. Cell nuclei were labeled with DAPI. Coverslips were mounted on slides with an anti-fading medium (Hydromount; National Diagnostics, Atlanta, GA), then imaged and analyzed as IHC above^12^. For cell viability assay, PC12 cells were seeded in 96-well plates at a density of 5 × 10^3^ cells/well. After reaching 80% confluence, cells were incubated with or without 50 μM Boc-D-FMK (Cayman Chemical Company, Cat. No. 16118), 30 mM necrostatin-1 (Cayman Chemical Company, Cat. No. 11658)^3^, 20μg/ml cycloheximide (Sigma-Aldrich, Cat. No. C7698), 100 μM chloroquine, 100 nM bafilomycin A, and recombinant rat TNFα (0, 25, 50 ng/ml BioLegend, Cat. No. 580102) for 18 h. Cell viability was analyzed by CellTiter-Glo Luminescnet Cell Viability Assay kit (Promega, Cat. No. G7570) following the manufacturer protocol^36^.

### Statistical analysis

Unless indicated otherwise, all in vivo results are expressed as mean ± SEM, where “*n*” is the number of individual animals per group. The number of animals in all studies was determined by power analysis (power of 0.8 with alpha value 0.05). Key experiments were repeated with independent groups of animals to ensure reproducibility. For in vitro cell based assays results are expressed as mean ± SD; “*n*” is the number of total replicates from at least three independent experiments. All statistical analyses were conducted using SigmaPlot, Version 12 (Systat Software, San Jose, CA) or GraphPad Prism, Version 3.02 for Windows (GraphPad Software, La Jolla, CA). One-way or two-way (cell death assay and in vivo rapamycin treatment) ANOVA followed by Bonferroni, Tukey’s or SNK *t*-test post-hoc test was used for parametric data. Kruskal–Wallis ANOVA based on ranks and Dunn’s post-hoc test was used for non-parametric data. For experiments with only two groups two-tailed unpaired Student’s *t*-test (parametric) was performed. A *p* value ≤ 0.05 was considered significant^12,16^.

## Electronic supplementary material

Supplementary Figures
