# Supplementary material for: Lysosomal damage after spinal cord injury causes accumulation of RIPK1 and RIPK3 proteins and potentiation of necroptosis
Source: Cell Death Dis. 2018 Apr 23;9(5):476. doi: 10.1038/s41419-018-0469-1 (PMC5913300; doi:10.1038/s41419-018-0469-1)
Supplement: Supplementary file 1 — Supplementary Figures [file 41419_2018_469_MOESM1_ESM.pdf]

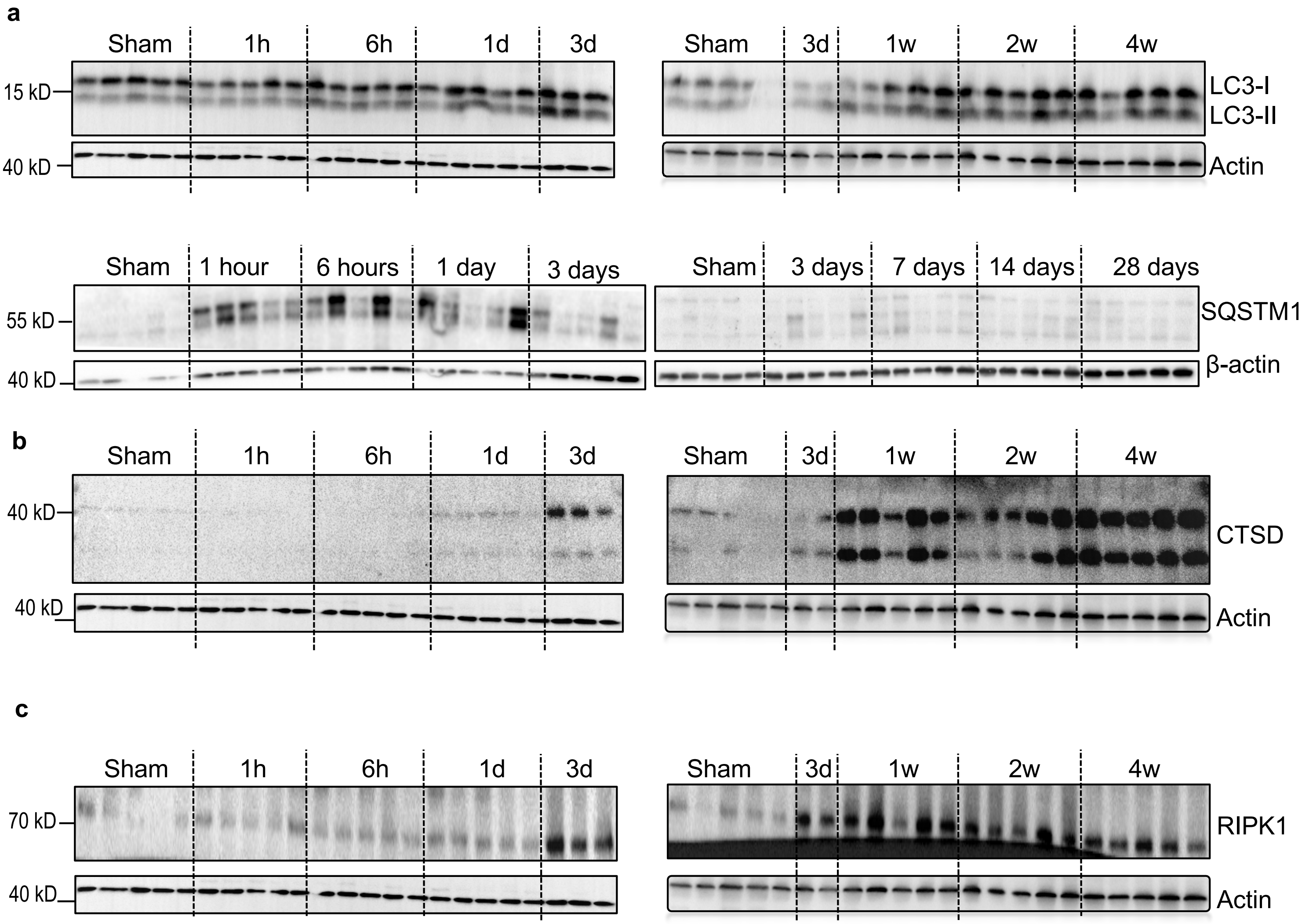

Supplementary Figure S1. Unedited SCI and sham control mouse western blots from paper. **a)** LC3 and p62/SQSTM1 blots from Figure 1a. **b)** CTSD blot from Figure 2a. **c)** RIPK1 blot from Figure 3a. C57Bl6 mice were subject to SCI or sham surgery. Spinal cord samples were collected at indicated time points, processed for western blot, and blotted with indicated antibodies. In each case loading control (actin) is from the same blot as the experimental sample.

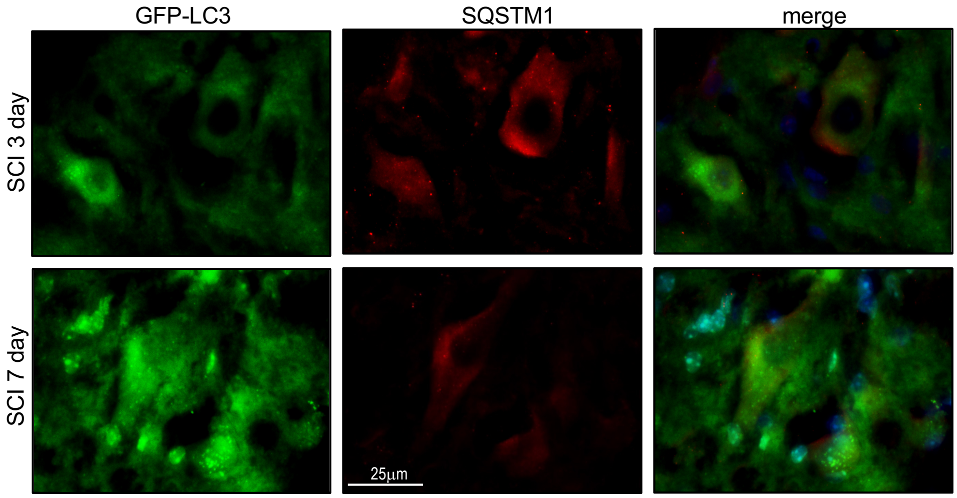

Supplementary Figure S2. Time course of expression of GFP-LC3 and p62/SQSTM1 in the spinal cord ventral horn after SCI. GFP-LC3 mice subject to SCI or sham surgery were perfused at indicated time points and processed for frozen sections (20μm). Sections were stained with antibodies against SQSTM1 and counterstained with DAPI to visualize nuclei. Images were acquired at 60x magnification.

a

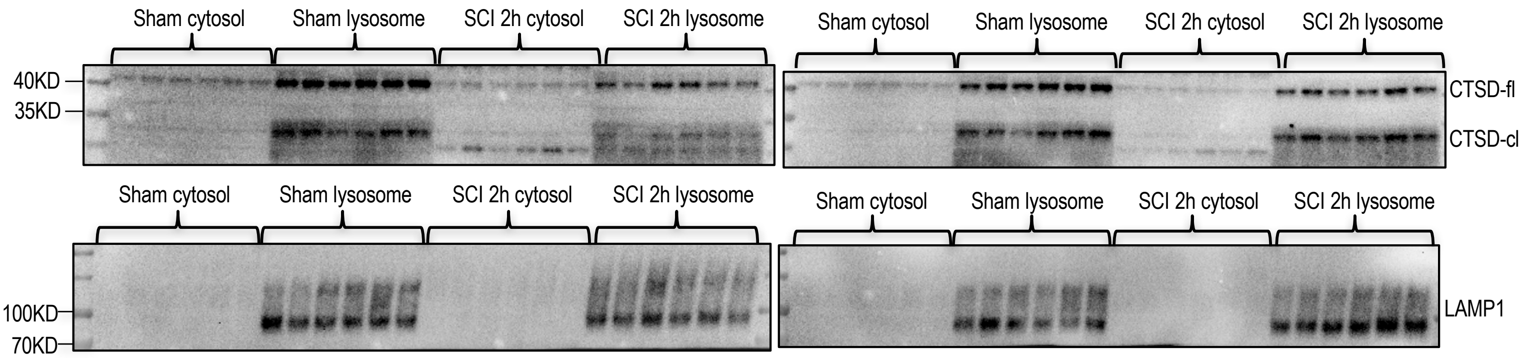

b

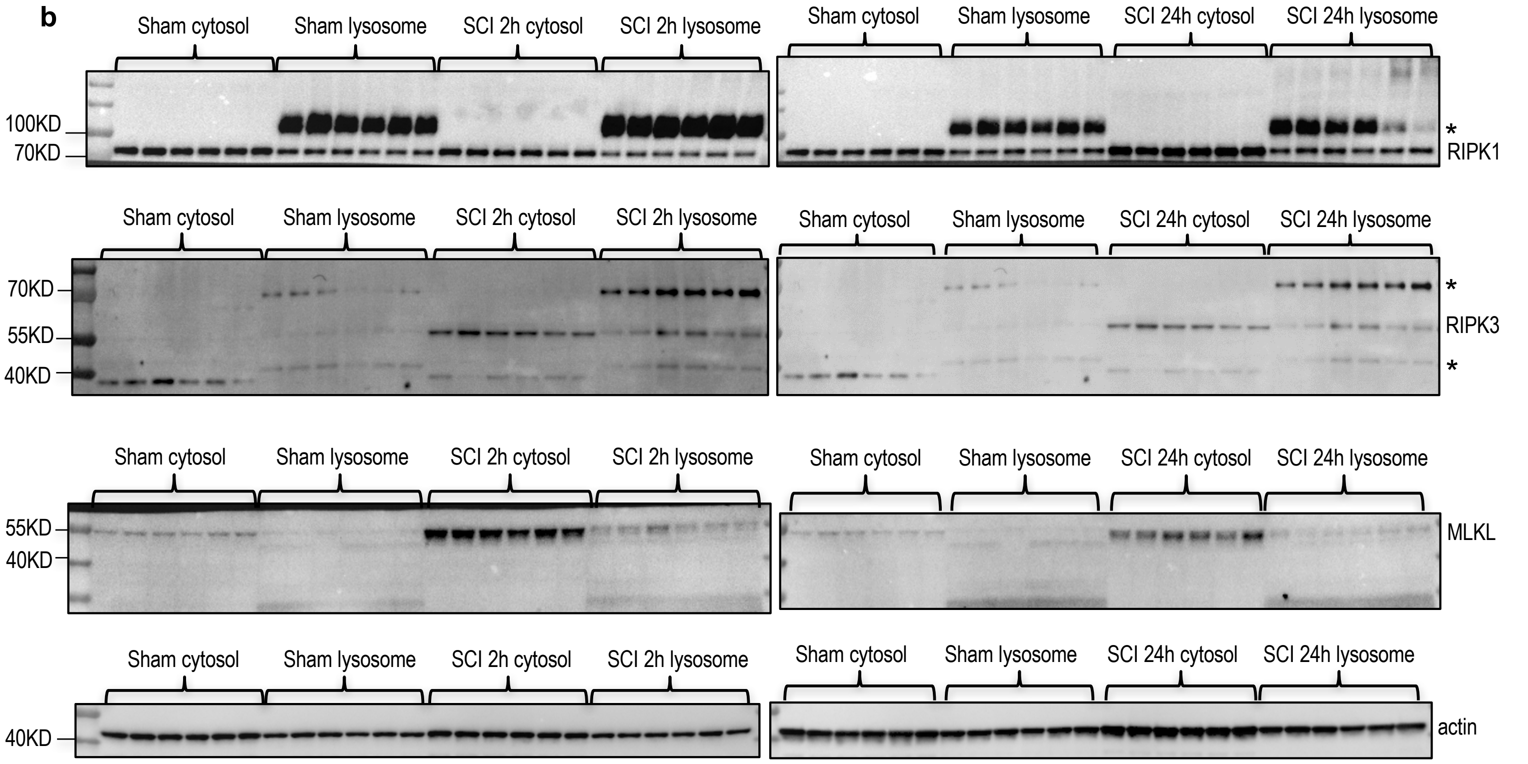

Supplementary Figure S3. Unedited western blots for the subcellular fractionation experiments in **a)** Figure 2c and **b)** Figure 6a. The same LAMP1 blot was used to assess fractionation in a and b. C57Bl6 mice were subject to SCI or sham surgery. Spinal cord samples were collected at indicated time points, fractionated to purify lysosome-enriched and cytosolic fractions, processed for western blot and blotted with indicated antibodies. *non-specific bands

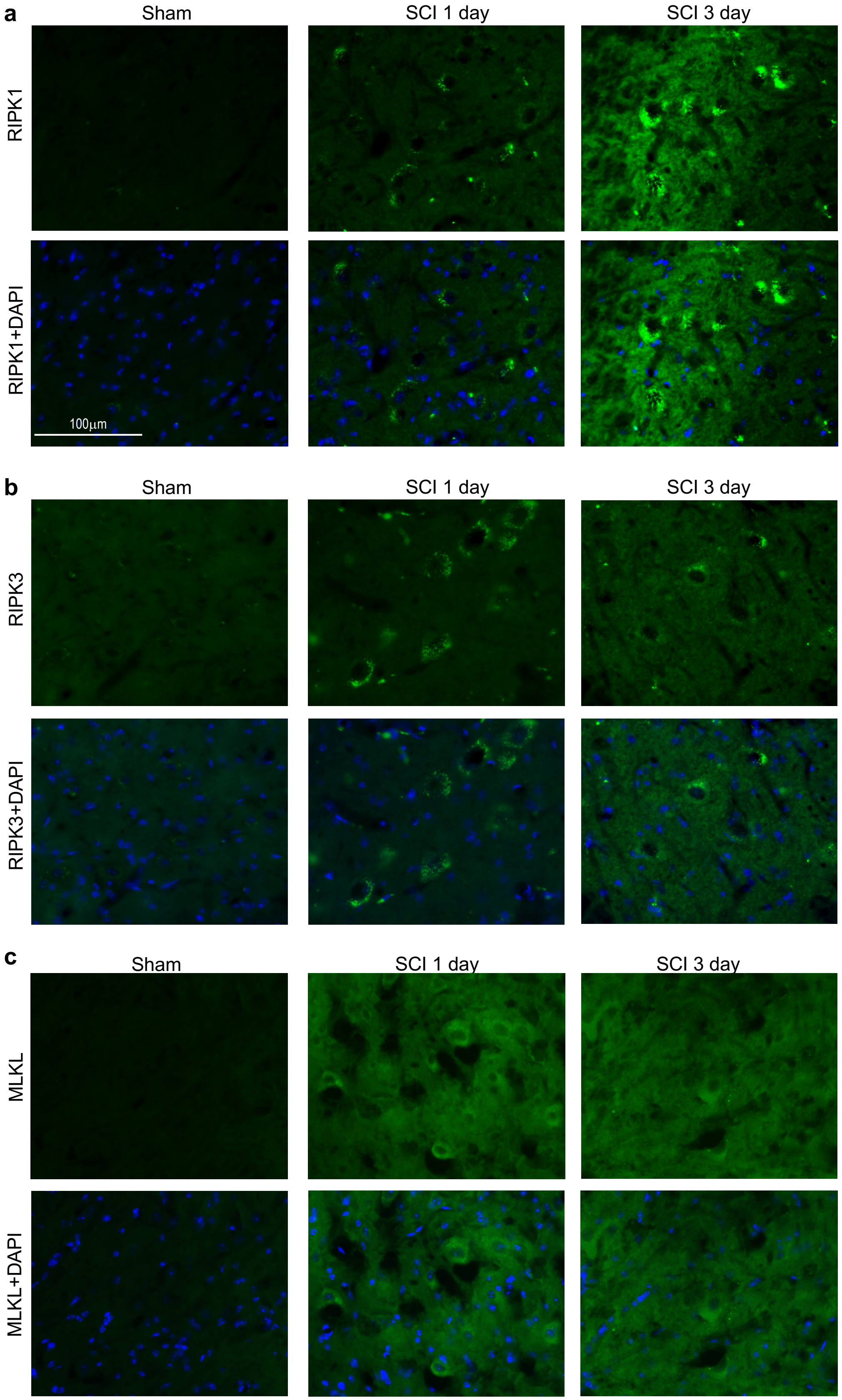

Supplementary Figure S4. Time course of expression of RIPK1, RIPK3 and MLKL in the spinal cord ventral horn after SCI. **a)** IHC for expression of RIPK1. Quantification of these data is presented in Figure 3d. **b)** IHC for expression of RIPK3. Quantification of these data is presented in Figure 4e. **c)** IHC for expression of MLKL. Quantification of these data is presented in Figure 4h. Mice subjected to SCI or sham surgery were perfused at indicated time points and processed for frozen sections (20µm). Sections were stained with antibodies against RIPK1, RIPK3 or MLKL and counterstained with DAPI to visualize nuclei. Images were acquired at 20x magnification.

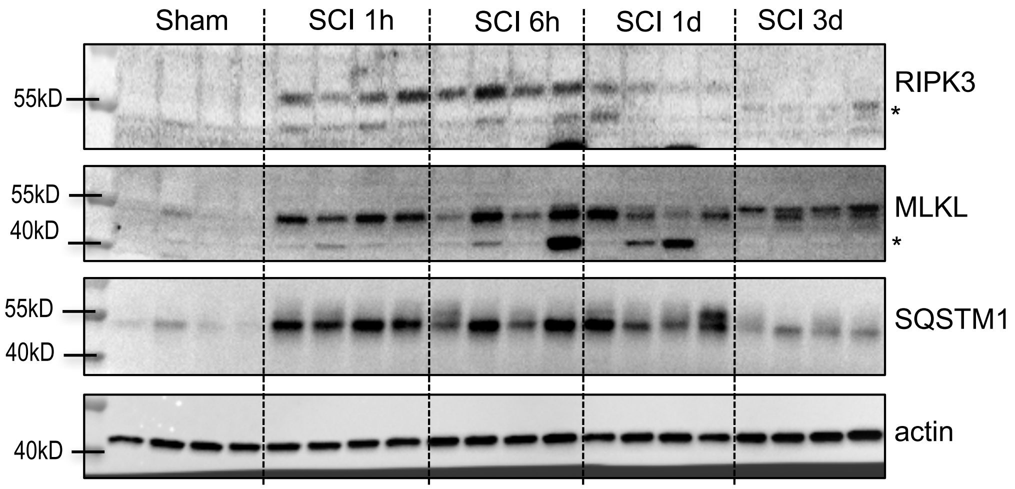

Supplementary Figure S5. Unedited RIPK3, MLKL and p62/SQSTM1 SCI and sham control mouse western blots from Figure 4a in the paper. C57Bl6 mice were subjected to SCI or sham surgery. Spinal cord samples were collected at indicated time points, processed for western blot, and blotted with indicated antibodies. Loading control (actin) is from the same blot as the experimental samples. * denotes non-specific bands.

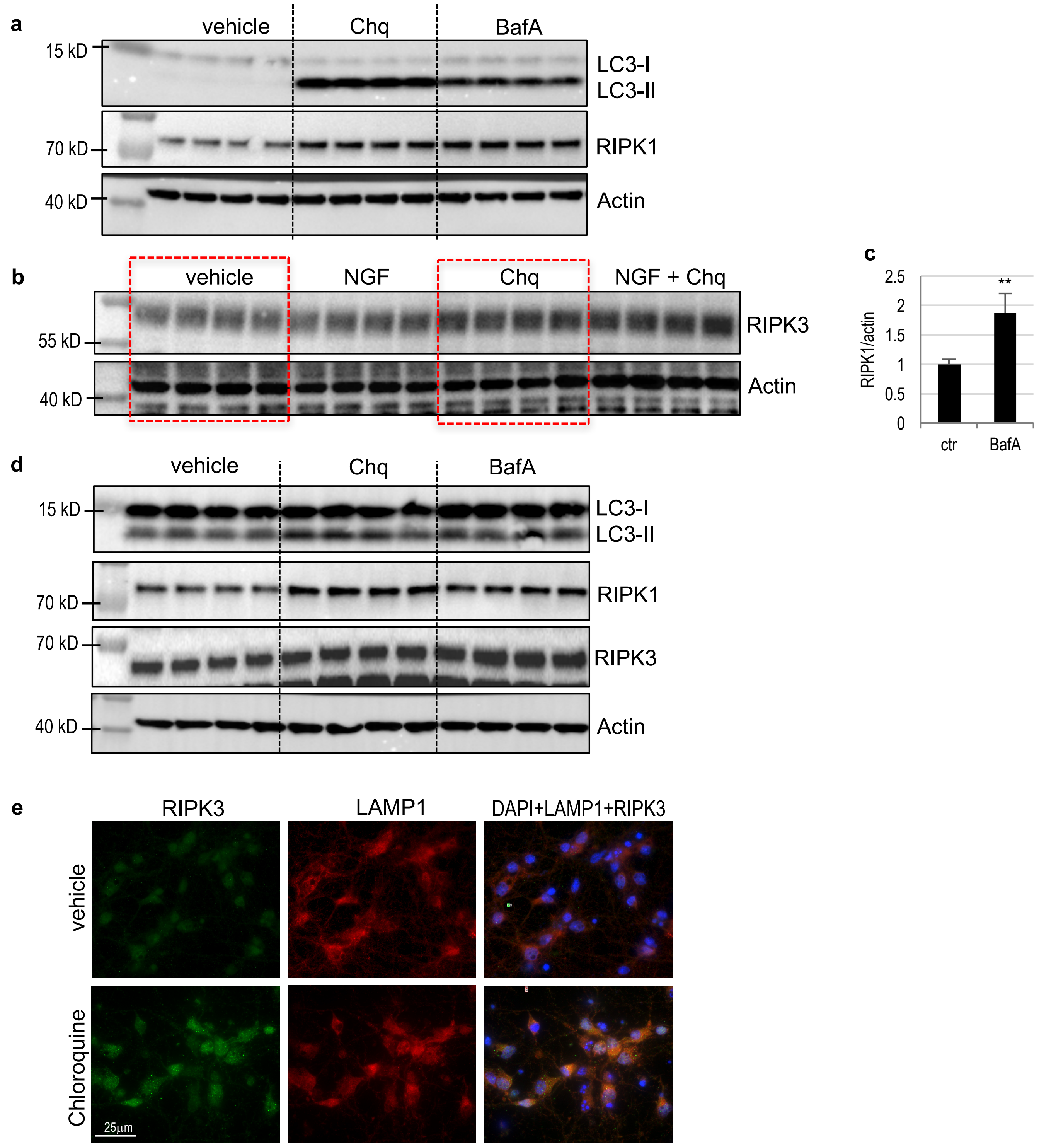

Supplementary Figure S6. Accumulation of RIPK1 and RIPK3 following lysosomal inhibition *in vitro*. **a-c)** Unedited western blots from Figure 5a and 5c in the paper. **a)** LC3 and RIPK1in PC12 cells blots from Figure 5a. **b)** RIPK3 in PC12 cells blot from Figure 5a. Levels of RIPK3 were assessed in the presence and absence of NGF. Sections framed in red are shown in the paper. **c)** Quantification of RIPK1 following BafA treatment shown in panel a. **p<0.01; graph represents mean +/- SD; n=4 **d)** LC3, RIPK1 and RIPK3 in rat cortical neurons blots from Figure 5d. All cells were treated for 4 hours, than processed for western blot. **e)** IF images demonstrating accumulation of RIPK3 in rat cortical neurons treated with Chq. Cells were treated for 4 hours, then fixed with PFA and processed for IF. Images were acquired at 20x magnification. Quantification of the data is shown in Figure 5i.

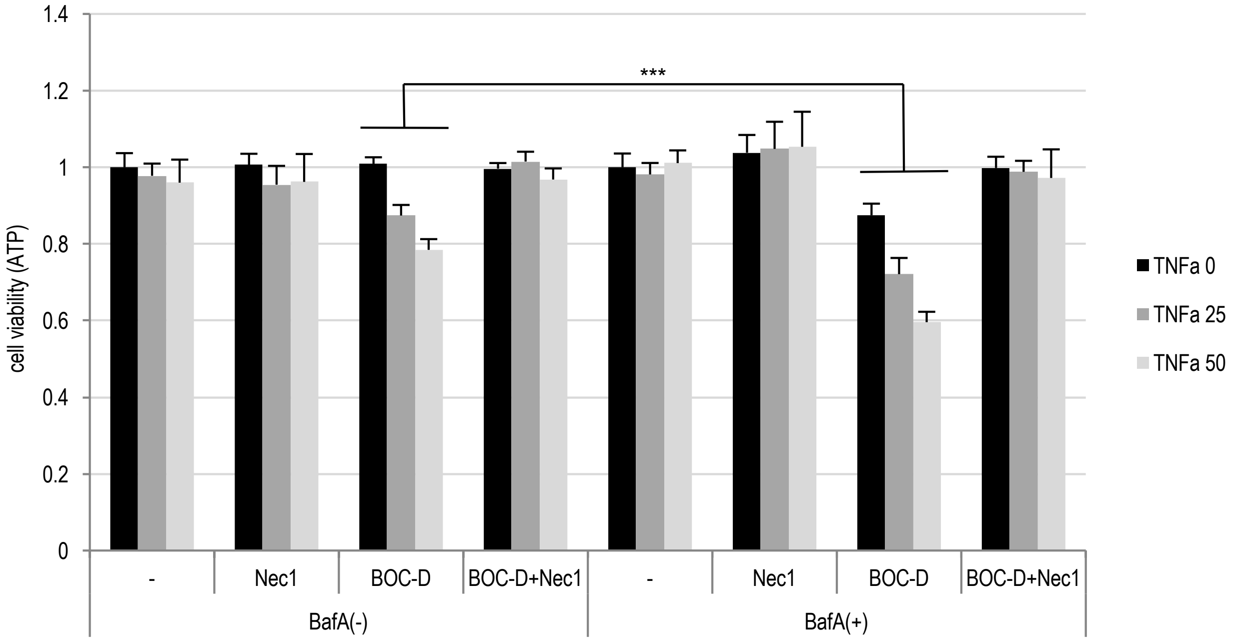

Supplementary Figure S7. Extended data including all controls from Figure 5k showing effects of lysosomal inhibition on PC12 cell viability. PC12 cells were treated in presence of cycloheximide (20μg/ml) with indicated doses of rat TNFα (0, 25, 50ng/ml) and pan-caspase inhibitor Boc-D (50μM) to induce either apoptosis (no Boc-D) or necroptosis (with Boc-D). BafA (100nM) was used to inhibit lysosomal function and RIPK1 inhibitor necrostatin 1 (Nec1, 30μM) was used to confirm that cell death was dependent on necroptosis. After 18 hours cell viability was measure using luminescent ATP assay. All data are presented as mean + SD. n=6 *$p<0.05$, **$p<0.01$, ***$p<0.001$

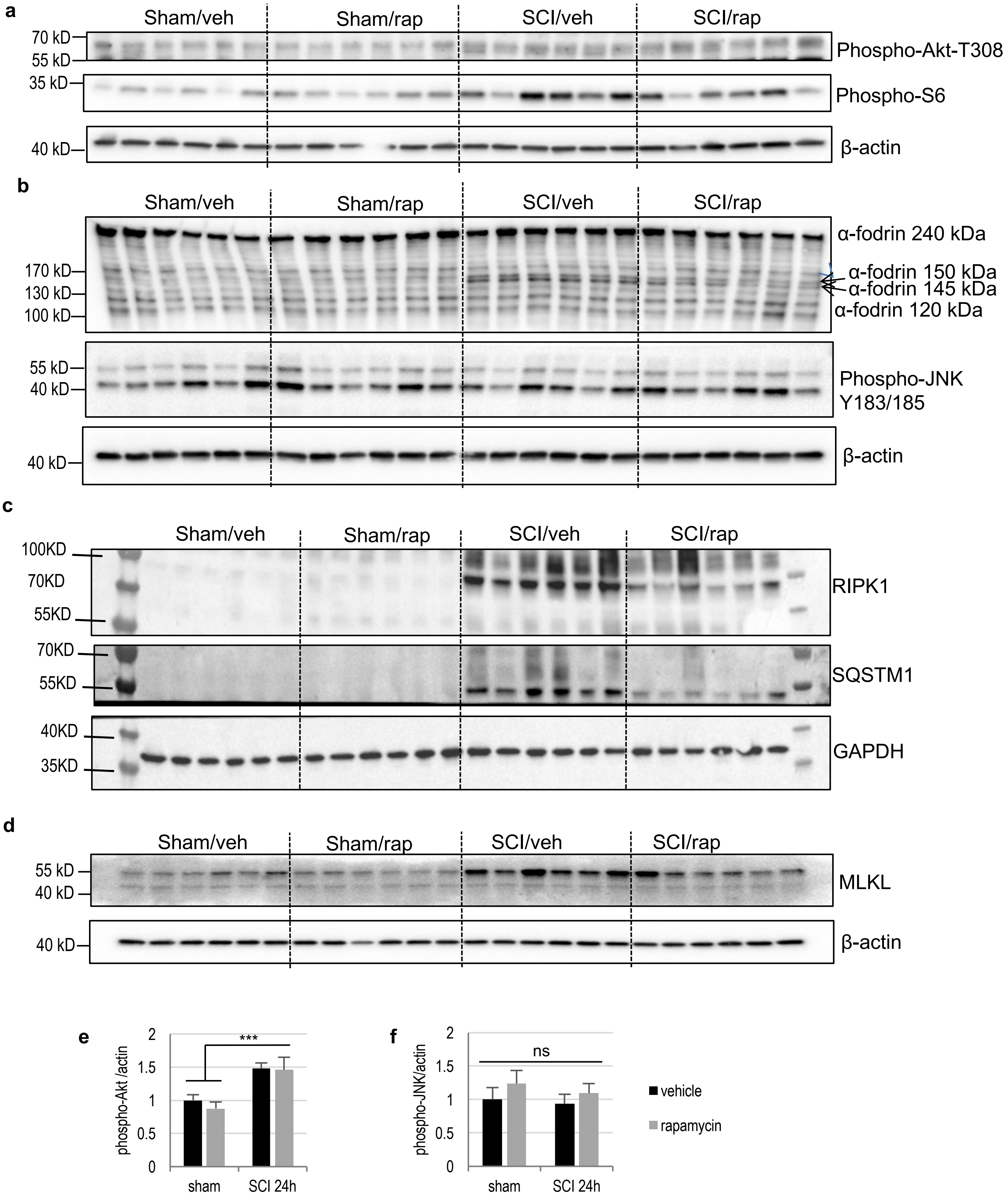

Supplementary Figure S8. Unedited western blots from Figure 6e in the paper. C57Bl6 mice were subjected to SCI or sham surgery followed by IP injection of 5 mg/Kg rapamycin or vehicle. Spinal cord samples were collected 24 hours after surgery. Loading control (GAPDH or actin) is from the same blot as the experimental samples. Additional blots demonstrating activity of the Akt (a) and JNK (b) pathways are also included. e) Quantification of phospho-Akt-T308 from a. f) Quantification of phospho-JNK-Y183/185 from b. Data are presented as mean +/- SE. n=6 *p<0.05, **p<0.01, ***p<0.001
